# Effectiveness of the Modified Vaccinia Ankara Vaccine Against Mpox in Men Who Have Sex With Men: A Retrospective Cohort Analysis, Seattle, Washington

**DOI:** 10.1093/ofid/ofad528

**Published:** 2023-10-24

**Authors:** Meena S Ramchandani, Anna Berzkalns, Chase A Cannon, Julia C Dombrowski, Elizabeth Brown, Eric J Chow, Elizabeth Barash, Sargis Pogosjans, Daniel Smith, Matthew R Golden

**Affiliations:** Department of Medicine, University of Washington, Seattle, Washington, USA; HIV/STI/HCV Program, Public Health–Seattle & King County, Seattle, Washington, USA; HIV/STI/HCV Program, Public Health–Seattle & King County, Seattle, Washington, USA; Department of Medicine, University of Washington, Seattle, Washington, USA; HIV/STI/HCV Program, Public Health–Seattle & King County, Seattle, Washington, USA; Department of Medicine, University of Washington, Seattle, Washington, USA; HIV/STI/HCV Program, Public Health–Seattle & King County, Seattle, Washington, USA; Department of Epidemiology, University of Washington, Seattle, Washington, USA; Department of Biostatistics, University of Washington, Seattle, Washington, USA; Department of Medicine, University of Washington, Seattle, Washington, USA; Department of Epidemiology, University of Washington, Seattle, Washington, USA; Communicable Disease Epidemiology and Immunizations, Public Health–Seattle & King County, Seattle, Washington, USA; HIV/STI/HCV Program, Public Health–Seattle & King County, Seattle, Washington, USA; Communicable Disease Epidemiology and Immunizations, Public Health–Seattle & King County, Seattle, Washington, USA; Communicable Disease Epidemiology and Immunizations, Public Health–Seattle & King County, Seattle, Washington, USA; Department of Medicine, University of Washington, Seattle, Washington, USA; HIV/STI/HCV Program, Public Health–Seattle & King County, Seattle, Washington, USA; Department of Epidemiology, University of Washington, Seattle, Washington, USA

**Keywords:** JYNNEOS, modified vaccinia Ankara, mpox, MPXV, MVA

## Abstract

**Background:**

Data on modified Vaccinia Ankara (MVA) vaccine effectiveness against mpox in real-world settings are limited.

**Methods:**

We performed a retrospective cohort analysis using Cox proportional hazards regression to estimate the association between vaccination and laboratory-confirmed mpox incidence. Study subjects included all men who have sex with men seen in a sexual health clinic in Seattle, Washington, between 1 January 2020 and 31 December 2022. Subjects’ receipt of vaccine and diagnosis with mpox were ascertained from public health vaccine registry and surveillance data. Analyses were adjusted for demographic factors, human immunodeficiency virus (HIV) status, and sexual risk behaviors.

**Results:**

The incidence of mpox per 100 person-years was 8.83 among patients with 0 doses, 3.32 among patients with 1 dose, and 0.78 among patients with 2 doses of MVA vaccine. Mpox diagnosis was significantly associated with age category 30–39 and 40–51 years, HIV positivity, syphilis diagnosis in the prior year, >10 sex partners in the last year, and having a clinic visit in the last year. In the multivariate model adjusting for these factors, vaccine effectiveness was 81% for 1 dose and 83% for 2 doses.

**Conclusions:**

These data support the effectiveness of the MVA vaccine—including a single dose of the vaccine—in preventing mpox disease and highlight the appropriateness of risk factor-based prioritization of immunization early in the epidemic. The durability of MVA vaccine-induced immunity is unknown, and at-risk persons should receive 2 doses of MVA.

## Teaser

In a retrospective cohort analysis of >4200 men who have sex with men attending a sexual health clinic, using Cox proportional hazards regression, vaccine effectiveness of modified vaccinia Ankara against mpox was similar for 1 or 2 doses at about 81%.

Between May 2022 and June 2023, >30 000 cases of confirmed mpox infection occurred in the United States (US) and >85 000 cases occurred globally, with the overwhelming majority of cases diagnosed among men who have sex with men (MSM). The modified vaccinia Ankara vaccine (MVA) (JYNNEOS, Bavarian Nordic) is approved by the US Food and Drug Administration (FDA) to prevent both smallpox and mpox and has been widely used during the current outbreak to prevent mpox disease.

Data on the efficacy of MVA against mpox in humans is limited. The vaccine was originally approved based on evidence of immunogenicity and data from animal challenge models [1, 2]. More recently, studies have produced somewhat variable estimates of vaccine effectiveness, with a single dose of vaccine reported to be 36%–86% effective in preventing mpox and 2 doses estimated to be 66%–89% effective [3–7]. We conducted a retrospective observational cohort study of the incidence of mpox disease among MSM seen in a sexual health clinic (SHC) in Seattle, Washington.

## METHODS

### Subjects

The study cohort included all Public Health–Seattle & King County (PHSKC) SHC patients who resided in King County, had at least 1 clinic visit between 1 January 2020 and 31 December 2022, and who were assigned male sex at birth and reported sex with men. Study subject characteristics, including sex assigned at birth, current gender, gender of sex partners, age, race, ethnicity, human immunodeficiency virus (HIV) status, methamphetamine use in the last year, and gonorrhea or syphilis diagnosis in the last year were abstracted from the SHC electronic medical record. Data collection procedures for the clinic have been previously described [8]. Based on previous data in Washington State, we have found that MSM were at the greatest risk of HIV diagnosis after being diagnosed with rectal gonorrhea and syphilis and prioritize these persons for sexually transmitted infection (STI) and HIV prevention [9, 10].

### Vaccine Eligibility and Timeline

Washington State received its first allocation of MVA vaccine in June 2022. Due to limited vaccine availability, this vaccine was initially provided for postexposure prophylaxis to close contacts exposed to mpox. Vaccine eligibility was modified when more vaccine became available. Based in part on prior studies evaluating factors associated with HIV acquisition in King County residents [10, 11], subsequent local guidelines adopted in July redefined the population eligible for immunization to include gay, bisexual, or other men who have sex with men (GBMSM) and transgender persons who have sex with men with at least 1 of the following risk criteria: >10 sex partners in the last 3 months, history of early syphilis or gonorrhea in the last year, methamphetamine use in the last month, attendance at a public sex venue or bathhouse or group sex in the past 3 months, or experienced homelessness/unstable housing or living in a congregate setting. PHSKC initially prioritized providing a first dose of MVA to as many eligible people as possible. On 9 August 2022, the FDA authorized emergency use of MVA by intradermal injection, which increased the total number doses available for use 3- to 4-fold [12], and PHSKC adopted intradermal injections as the primary mode of administration. On 22 August 2022, the SHC and other local providers began to provide second doses of MVA. Vaccine eligibility criteria at that time were expanded to include sex workers of any sexual orientation or gender identity, GBMSM and transgender persons who have sex with men with multiple or anonymous sex partners or incarceration in the last 3 months, and GBMSM and transgender persons who have sex with men and who identified as a person of color. In the US, it recognized early on in the epidemic that persons of color were disproportionately affected by mpox infections but were less likely to receive the mpox vaccine. To help improve these disparities, prioritization of mpox vaccine was given to persons of color and people at higher risk of being exposed to mpox [13].

## Vaccination Status and Mpox Diagnosis

Ascertainment of vaccination status was based on data in the SHC medical record and matching of study subjects to MVA immunization data from the Washington State Immunization Information System (WAIIS) for vaccinations administered through 31 December 2022. It was required for MVA immunization to be reported to WAIIS for persons in Washington State. Mpox diagnosis status was based on matching of study subjects to public health mpox surveillance data and included diagnoses through 31 December 2022. In King County, mpox is an immediately notifiable condition. Clinicians and laboratories are required to report suspected and confirmed mpox cases to PHSKC. In October 2022, SHC began offering asymptomatic mpox screening to MSM patients; positive results identified through this screening were not included as incident mpox cases in the analysis. We used Link Plus, a probabilistic record linkage program developed at the Centers for Disease Control and Prevention (CDC), to link SHC patient data to mpox case surveillance data and WAIIS [14]. Link Plus was used to merge information from 2 sources to the SHC patient cohort to identify cases diagnosed with other providers. A cutoff value of 8 for Link Plus was used during the matching process. All matches with a score >8 were then manually reviewed to verify if a true match. All others were assumed to be unvaccinated.

## Statistical Analysis

We calculated the incidence of mpox, including person-time and number of events, in those who had received 0, 1, or 2 doses of MVA. We used vaccination date plus 14 days to create a vaccination effective date, and treated vaccination status (0, 1, or 2 doses) as a time-dependent covariate in Cox proportional hazards models. Vaccine effectiveness was estimated as 1 minus the hazard ratio for 1 and 2 doses versus 0 doses. All patients began follow-up on 1 May 2022 and patients were censored at mpox diagnosis or 31 December 2022. We calculated vaccine effectiveness as 1 minus the estimated hazard ratio. The Cox model was adjusted for age, race/ethnicity, HIV status, year of last SHC visit, methamphetamine use in the last year, gonorrhea or syphilis diagnosis in the last year, and self-report of >10 sex partners in the last year; apart from age, these variables were based on medical chart data abstracted from the patient's most recent SHC visit prior to 31 December 2022. We found each of these covariates were significant in the bivariate or unadjusted model except for race/ethnicity. Analyses were completed in SAS version 9.4 and R version 4.1 software. We did not stratify on route of administration and subcutaneous and intradermal routes of administration were treated equally for the purposes of this analysis.

The study was exempt from institutional review board approval as it was part of public health surveillance and program evaluation.

## RESULTS

A total of 4230 MSM attended the PHSKC SHC from 1 January 2020 to 31 December 2022 (Table 1). The median age was 34 years. Sixty-one percent were White, 11% Black or African American, 10% Asian, and 18% Hispanic or Latino. Six percent of participants had used methamphetamine in the last year, 15% were HIV positive, and 33% had gonorrhea and 13% had syphilis in the last year. Approximately 34% of patients were currently using HIV preexposure prophylaxis and 23% had >10 sex partners in the last year.

**Table 1.** Characteristics of 4230 Men Who Have Sex With Men Attending the Public Health–Seattle & King County Sexual Health Clinic From 1 January 2020 to 31 December 2022

Characteristic	No. (%)
Age group, y	
Median	34.0
<30	1250 (30)
30–39	1681 (40)
40–51	769 (18)
≥52	530 (13)
Race	
White	2569 (61)
Black or African American	451 (11)
Asian	417 (10)
Multiracial or other	100 (2)
American Indian or Alaska Native	39 (<1)
Pacific Islander or Native Hawaiian	32 (<1)
Unknown	622 (15)
Ethnicity	
Hispanic or Latino	768 (18)
Non-Hispanic	3303 (78)
Unknown	159 (4)
Methamphetamine use in the last year	
Yes	264 (6)
No	3180 (75)
Unknown	786 (19)
HIV status	
Positive	630 (15)
Negative	3085 (73)
Unknown	515 (12)
Gonorrhea in the last year	
Yes	1379 (33)
No	2200 (52)
Unknown	651 (15)
Syphilis in the last year	
Yes	531 (13)
No	2976 (70)
Unknown	723 (17)
>10 sex partners in the last year	
Yes	961 (23)
No	2363 (56)
Unknown	906 (23)
Vaccination status as of 31 Dec 2022	
0 doses	2393 (57)
1 dose	685 (16)
2 doses	1152 (27)

Abbreviation: HIV, human immunodeficiency virus.

**Figure 1.**
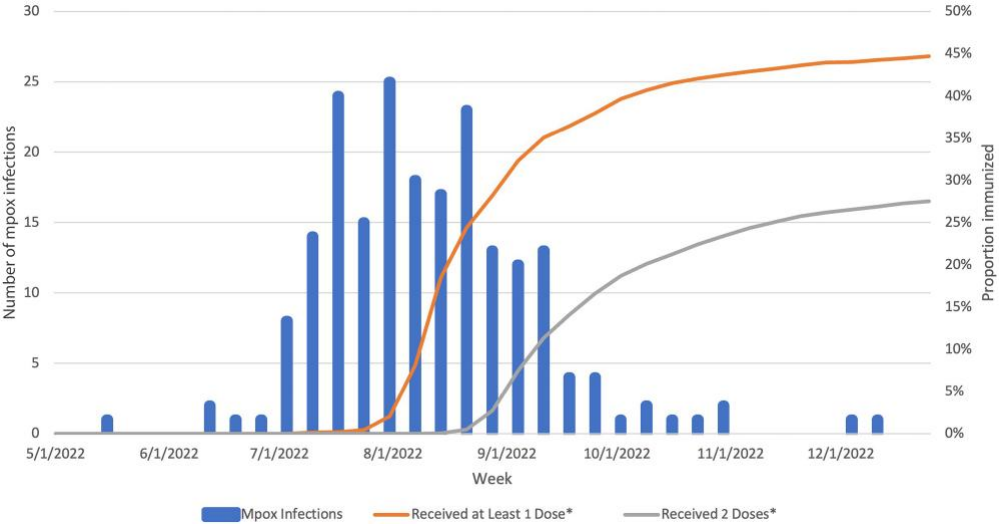
Mpox diagnoses per week and percentage of patients immunized against mpox among men who have sex with men attending a sexual health clinic by week—Seattle, Washington, May–December 2022. *Immunized defined as immunization date plus 14 days.

Among cohort members between 1 May 2022 and 31 December 2022, a total of 204 mpox infections were diagnosed, including 191 cases in people who received 0 doses of vaccine, 11 cases in people who received 1 dose of vaccine, and 2 cases in people who had received 2 doses of vaccine. The majority of mpox cases were diagnosed in July to September 2022 (Figure 1). Among patients diagnosed with mpox after 1 dose of vaccine, the mean time from vaccine-effective date to diagnosis date was 37.3 days and the median was 31 (interquartile range, 19–39) days. The 2 patients diagnosed after 2 doses of vaccine were diagnosed 44 and 82 days after the second vaccine-effective date. The incidence of mpox was 8.83 per 100 person-years among patients with 0 doses of vaccine, 3.32 per 100 person-years among patients with 1 dose of vaccine, and 0.78 per 100 person-years among patients with 2 doses of vaccine. In the unadjusted model, compared to receipt of no vaccine, 1 dose was 60% effective and 2 doses 57% effective in preventing laboratory-confirmed mpox (Table 2); the hazard ratio for 2 doses was not statistically significant.

**Table 2.** Cox Proportional Hazards and JYNNEOS Vaccine Effectiveness Estimates

Characteristic	Mpox Diagnoses	Patients	Unadjusted			Adjusted^a^		
	No.	No.	HR (95% CI)	Estimated Effectiveness	*P* Value	HR (95% CI)	Estimated Effectiveness	*P* Value
Vaccine doses								
0	191	2393	(Ref)	(Ref)	(Ref)	(Ref)	(Ref)	(Ref)
1	11	685	0.40 (.21–.76)	60%	.005*	0.19 (.10–.36)	81%	<.001*
2	2	1152	0.43 (.10–1.84)	57%	.256	0.17 (.04–.72)	83%	.016*
Age, y								
<30	41	1250	0.91 (.53–1.57)		.745	1.38 (.78–2.42)		.264
30–39	88	1681	1.47 (.90–2.42)		.125	1.96 (1.18–3.26)		.009*
40–51	56	769	2.07 (1.23–3.49)		.006*	2.31 (1.37–3.90)		.002*
≥52	19	530	(Ref)		(Ref)	(Ref)		(Ref)
Race								
White	113	2182	(Ref)		(Ref)	(Ref)		(Ref)
American Indian or Alaska Native	3	22	2.66 (.84–8.37)		.095	2.12 (.67–6.72)		.201
Asian	12	405	0.56 (.31–1.02)		.059	0.73 (.40–1.34)		.309
Black or African American	13	421	0.59 (.33–1.04)		.068	0.62 (.35–1.11)		.109
Hispanic/Latinx	51	924	1.07 (.77–1.48)		.703	0.92 (.66–1.30)		.643
Pacific Islander or Native Hawaiian	0	27	0.0 (.0–∞)		.993	0.0 (.0–∞)		.989
Multiracial or other	6	81	1.43 (.63–3.26)		.391	1.47 (.64–3.38)		.363
Unknown	6	168	0.69 (.30–1.57)		.374	0.80 (.35–1.83)		.593
Methamphetamine use in the last year								
Yes	19	264	2.87 (1.74–4.73)		<.001*	1.59 (.92–2.76)		.095
No	81	3180	(Ref)		(Ref)	(Ref)		(Ref)
Unknown	104	786	5.51 (4.12–7.37)		<.001*	1.77 (.67–4.66)		.246
HIV status								
Positive	56	630	3.06 (2.20–4.27)		<.001*	2.90 (1.94–4.34)		<.001*
Negative	92	3085	(Ref)		(Ref)	(Ref)		(Ref)
Unknown	56	515	3.79 (2.72–5.28)		<.001*	0.98 (.65–1.49)		.676
Gonorrhea in the last year								
Yes	68	1379	2.50 (1.71–3.65)		<.001*	1.13 (.72–1.77)		.608
No	44	2200	(Ref)		(Ref)	(Ref)		(Ref)
Unknown	92	651	7.57 (5.28–10.84)		<.001*	1.90 (.89–4.03)		.097
Syphilis in the last year								
Yes	40	531	3.38 (2.28–4.99)		<.001*	1.90 (1.19–3.03)		.007*
No	68	2976	(Ref)		(Ref)	(Ref)		(Ref)
Unknown	96	723	6.19 (4.53–8.44)		<.001*	1.94 (.82–4.60)		.133
>10 sex partners in the last year								
Yes	56	961	2.80 (1.91–4.10)		<.001*	2.22 (1.49–3.30)		<.001*
No	50	2363	(Ref)		(Ref)	(Ref)		(Ref)
Unknown	98	906	5.36 (3.81–7.54)		<.001*	1.64 (1.08–2.51)		.021*
Year of last clinic visit								
2020	11	791	(Ref)		(Ref)	(Ref)		(Ref)
2021	11	1007	0.78 (.34–1.81)		.567	1.05 (.46–2.44)		.902
2022	182	2432	5.56 (3.02–10.21)		<.001*	6.02 (3.25–11.17)		<.001*

Abbreviations: CI, confidence interval; HIV, human immunodeficiency virus: HR, hazard ratio.

^a^Adjusted for age group, race/ethnicity, HIV status, year of last sexual health clinic visit, methamphetamine in the last year, gonorrhea diagnosis in the last year, syphilis diagnosis in the last year, and >10 sex partners in the last year.

^*^ *P* < .05.

In the adjusted multivariable analysis, mpox diagnosis was significantly associated with age group (30–39 and 40–51 years), HIV positivity, syphilis in the last year, >10 sex partners in the last year, and more recent year of last clinic visit (Table 2). Controlling for factors that were associated with mpox diagnosis in the unadjusted analysis, which included age group, race/ethnicity, HIV status, year of last SHC visit, methamphetamine use in the last year, >10 sex partners in the last year, and gonorrhea or syphilis diagnosis in the last year, vaccine effectiveness was 81% for 1 dose and 83% for 2 doses.

For those who received 1 dose of MVA, 216 received the vaccine subcutaneously and 469 intradermally. For those who received 2 doses of MVA, 36 received the vaccine subcutaneously, 434 received it intradermally, and 682 received 1 subcutaneous and 1 intradermal route of administration. There were 4 (1.9%) mpox cases in 215 patients who received 1 dose of vaccine subcutaneously, 7 (1.5%) cases in 468 patients who received 1 dose intradermally, and 2 (0.3%) cases in 681 patients who received 1 dose of vaccine subcutaneously and 1 dose intradermally. There were zero cases in patients who received 2 doses subcutaneously or 2 doses intradermally.

## DISCUSSION

We evaluated the effectiveness of the MVA vaccine to protect against mpox in >4200 MSM attending the SHC in Seattle, WA. The incidence of mpox per 100 person-years was 8.83 among patients with 0 doses, 3.32 among patients with 1 dose, and 0.78 among patients with 2 doses of MVA vaccine. In the adjusted multivariable model, vaccine effectiveness was similar for 1 or 2 doses at about 83%.

Other recently published studies have also demonstrated MVA protects against mpox disease in real-world settings, though the estimated vaccine effectiveness has varied. The reasons for this variance are uncertain but may reflect differences in study design and/or the extent to which the studies controlled for potential confounding. Three of the 5 studies evaluating MVA vaccine effectiveness employed case-control designs [4–6]; these studies reported vaccine effectiveness estimates of 36%–75% for 1 dose and 66%–89% for 2 doses of MVA vaccine. In contrast, a retrospective cohort study conducted in Israel estimated that 1 dose of vaccine was 86% effective, similar to findings in this study [7]. The vaccine effectiveness of 83% we found in our analysis is also similar to studies estimating vaccine effectiveness of 81%–85% of previous smallpox vaccination (vaccinia) to protect against mpox in the Democratic Republic of Congo from 2005 to 2007 and Zaire from 1980 to 1984 [15, 16].

While the case-control studies all sought to control for time through the choice of controls, this approach may not adequately control for variation in the incidence of mpox over time, potentially leading to higher estimates of second dose efficacy since second doses were typically administered later in the epidemic after incidence (and risk) had substantially declined. By using a Cox proportional hazards regression model aligned on calendar time to evaluate a large cohort of individuals, we were able to ensure this method controlled for time and comparisons were not confounded by changes in the underlying epidemic curve and the decline in the epidemic in the fall of 2022. Our model was able to account for the variation of incidence of mpox over time, when the majority of infections occurred prior to the second dose of vaccine. Only 1 study used a retrospective cohort design but was conducted early in the epidemic, observing only 21 mpox cases, and was not able to evaluate the vaccine effectiveness of 2 doses of the vaccine [7]. However, despite these limitations, this study also reported a vaccine effectiveness of 86% with a single dose of vaccine, similar to what was found in our study.

It is also possible that confounding factors other than time may have affected estimates of vaccine effectiveness. While all of the previously published studies controlled for some demographic factors and several controlled for factors such as history of STI [4, 6, 7], some did not [5]. The variable ability to control for such factors and selection of controls in a case-control design may have influenced estimates of vaccine effectiveness.

Our data should not be interpreted to suggest that persons at risk for mpox should forgo a second dose of MVA vaccine. The durability of vaccine-induced immunity following a single or even 2 doses of MVA vaccine is unknown. As of May 2023, the Illinois Department of Public Health reported increases of mpox cases in Chicago and Cook County, and CDC continues to report a small number of new mpox cases occurring in the US on an almost daily basis [17, 18]. Given these realities, we believe that medical providers and public health authorities should continue to recommend that patients receive a second dose of vaccine. At the same time, we are encouraged that 1 dose of vaccine seems to provide substantial protection, at least in the short term.

The analysis we present, as well as data from other mpox vaccine effectiveness studies, supports the risk-based vaccine prioritization system that PHSKC and many other health departments adopted early in the mpox epidemic when the supply of vaccine was inadequate. In particular, we found that history of syphilis and >10 sex partners—both of which were criteria for prioritizing vaccine—were significantly associated with mpox diagnosis. The fact that >98% of all mpox cases in King County occurred in GBMSM, transgender, and nonbinary persons likewise supports the initial approach to prioritizing vaccine, which was necessary given limited vaccine supply early in the outbreak response. We included year of last SHC visit in our analysis because we hypothesized that some patients may have left King County and consequently would not be at risk for mpox diagnosis or vaccination as measured by our database. We observed such an association supporting our hypothesis.

Our results support that the intradermal administration of MVA was protective against mpox infection. While we were not able to provide a statistical analysis of different administration methods, there was no indication from our experience that patients were less effectively protected by this alternative route of administration. Only 2 cases of mpox occurred in persons who received 1 dose of subcutaneous and 1 dose of intradermal vaccine and zero cases occurred in persons who received 2 doses of intradermal vaccine. The efficacy of intradermal administration of the MVA vaccine is also supported by immunogenicity data and other recently published studies [4, 5, 12, 19, 20].

Our study has both strengths and limitations. Strengths include our cohort design, which allowed us to control for time and associated variability in the incidence of mpox over the course of the study period; our ability to control for factors that have consistently been associated with incident STIs in MSM, including history of STI and methamphetamine use; and our use of population-level surveillance data to define vaccination status and mpox diagnoses. Important limitations to our analysis include the potential for residual confounding; missingness of data for some adjustment variables; the fact that, while the question of HIV status is routinely asked of all patients via the computer-assisted self-interview, it is possible some patients were not willing to disclose status; incomplete ascertainment of vaccine status and mpox diagnoses occurring outside of Washington State; and the possibility that some people included in the study cohort may have left the area and consequently not been at risk for mpox in King County. Also, we could not account for smallpox vaccination occurring in the remote past, though the majority of mpox cases in King County occurred in individuals born after cessation of routine smallpox vaccination in the US, which ended in the early 1970s. Our use of historical data to define subjects’ sexual risk and network factors is imperfect; however, our group has previously evaluated the extent to which sexual risk behavior persist over time [11].

Using a retrospective cohort design, we found that the MVA vaccine was 83% effective in preventing mpox after adjusting for patient risk factors. We did not observe a difference in vaccine effectiveness between 1 or 2 doses of vaccine. These data lend further support for the real-world effectiveness of the vaccine in preventing mpox disease. Additional data are needed on the duration of protection provided by 1 and 2 doses of MVA vaccine. In the absence of such data, we support continuing to provide patients at high risk for mpox with 2 doses of vaccine.

## Notes

***Author contributions.*** All authors confirmed they have contributed to the intellectual content of this paper and have met the following 3 requirements: (1) substantial contributions to conception and design, or acquisition of data, or analysis and interpretation of data; (2) drafting the paper or revising it critically for important intellectual content; and (3) final approval of the version to be published.

***Acknowledgments.*** We acknowledge the outstanding work of the staff in the Public Health–Seattle & King County (PHSKC) Sexual Health Clinic and Communicable Disease case investigators for their excellent care of patients and rapid compassionate response to the mpox outbreak. We are grateful for the patients in our program.

***Patient consent.*** This study does not include factors necessitating patient consent. The study was part of PHSKC surveillance and program evaluation.

***Financial support.*** Research reported in this publication was supported by the National Institute of Allergy and Infectious Diseases, National Cancer Institute, National Institute of Mental Health, National Institute on Drug Abuse, National Institute of Child Health and Human Development, National Heart, Lung, and Blood Institute, National Institute on Aging, National Institute of General Medical Sciences, and National Institute of Diabetes and Digestive and Kidney Diseases, all of the National Institutes of Health (award number AI027757).
